# Anxiety in mothers of children with COVID-19 based on the State-Trait Anxiety Inventory

**DOI:** 10.1590/0034-7167-2024-0643

**Published:** 2026-07-31

**Authors:** Nagila Lima Fontenele, Daisyanne Augusto de Sales Santos, Isabela Araújo Linhares Castro, Felipe Moreira de Paiva, Fabíola Chaves Fontoura, Natalia Rodrigues Oliveira, Rhaiany Kelly Lopes de Oliveira, Maria Vera Lúcia Moreira Leitão Cardoso

**Affiliations:** I Centro Universitário Christus, Unichristus. Fortaleza, Ceará, Brazil; II Universidade Federal do Ceará. Fortaleza, Ceará, Brazil; III Hospital Regional Tarcísio de Vasconcelos Maia. Mossoró, Rio Grande do Norte, Brazil; IV Universidade Federal do Rio Grande, Hospital Universitário, Empresa Brazileira de Serviços Hospitalares. Rio Grande, Rio Grande do Sul, Brazil

**Keywords:** Anxiety, Mothers, COVID-19, Child, Nursing., Ansiedad, Madres, COVID-19, Niño, Enfermería.

## Abstract

**Objectives:**

to assess the anxiety level of mothers of children who had COVID-19 in a city in northeastern Brazil using the State-Trait Anxiety Inventory.

**Methods:**

this cross-sectional study included mothers of children with COVID-19 in their first year of life, reported in e-SUS Notifica from February 2020 to January 2021. Data collection was conducted via telephone contact, using a questionnaire on maternal sociodemographic and epidemiological variables, as well as the State-Trait Anxiety Inventory. The Fisher-Freeman-Halton exact test was used to assess associations between variables.

**Results:**

maternal anxiety was moderate (percentile between 25 and 75) for both the Trait Anxiety Inventory (50%) and the State Anxiety Inventory (50%). There was statistical significance for elevated anxiety among education level (p=0.010), income (p=0.027), and the State Anxiety Inventory.

**Conclusions:**

mothers with children confirmed to have COVID-19 presented moderate anxiety levels, with elevated levels in those with low education and low income.

## RESUMEN

**Objetivos:**

evaluar el nivel de ansiedad de las madres de niños con COVID-19 en una ciudad del noreste de Brasil mediante el Inventario de Ansiedad Estado-Rasgo.

**Métodos:**

este estudio transversal incluyó a madres de niños con COVID-19 en su primer año de vida, registrados en el e-SUS Notifica entre febrero de 2020 y enero de 2021. La recolección de datos se realizó mediante contacto telefónico, utilizando un cuestionario sobre variables sociodemográficas y epidemiológicas maternas, así como el Inventario de Ansiedad Estado-Rasgo. Se utilizó la prueba exacta de Fisher-Freeman-Halton para evaluar las asociaciones entre las variables.

**Resultados:**

la ansiedad materna fue moderada (percentil entre 25 y 75) tanto para el Inventario de Ansiedad Rasgo (50%) como para el Inventario de Ansiedad Estado (50%). Se observó significancia estadística para la ansiedad elevada según el nivel educativo (p=0,010), los ingresos (p=0,027) y el Inventario de Ansiedad Estado.

**Conclusiones:**

las madres con hijos con COVID-19 confirmado presentaron niveles moderados de ansiedad, con niveles elevados en aquellas con bajo nivel educativo y bajos ingresos.

## INTRODUCTION

COVID-19, the infection caused by the SARS-CoV-2 coronavirus, responsible for Severe Acute Respiratory Syndrome^(1)^, spread rapidly throughout the world. In addition to the immediate effects related to clinical symptoms and the risk of death, the pandemic caused persistent consequences, intensified by long periods of social isolation. These factors triggered various disorders in the population, with economic, social^(2)^ and, especially, mental health repercussions^(3)^. According to data from the World Health Organization^(4)^, there has been a 25% increase in cases of anxiety symptoms in several countries, affecting women more significantly, who have proven to be particularly susceptible to developing mental disorders during this period.

Questions about the impacts of COVID-19 on maternal and neonatal health have further increased anxiety during pregnancy. In South Korea, not only anxiety, but also symptoms of depression, post-traumatic stress disorder, and suicidal ideation were significantly higher in infected pregnant women^(5)^.

Becoming a mother makes managing physical and mental health increasingly challenging and stressful, contributing to worsening anxiety symptoms. This was evidenced in a study conducted in Israel, which showed that approximately 80% of the mothers surveyed reported mild to high anxiety levels due to COVID-19^(6)^. Furthermore, the pandemic has exacerbated the impact on the bond between mothers and children, particularly regarding baby care and emotional attachment^(7)^.

The anxiety levels experienced by mothers can be even higher when their child is diagnosed with COVID-19. A study conducted with parents of children suspected of having COVID-19 at an outpatient clinic of a tertiary hospital in Ankara, Turkey, showed moderate anxiety levels among parents and a high level of fear regarding virus transmission^(8)^. Another study, conducted at the University of Nebraska Medical Center, carried out an observational cohort study with 703 mothers and revealed that rates of maternal anxiety, depression, use of medication for psychiatric conditions, and a history of postpartum depression increased significantly during COVID-19, considering births between March 2020 and December 2021. These rates remained elevated after this period compared to pre-pandemic levels^(9)^.

Compromised mental health can have immediate and long-term consequences for mothers and their babies, highlighting the urgent need for targeted care and interventions^(10)^. Therefore, considering the importance of the maternal role in children’s well-being, anxiety assessment is a preventive and protective measure for women that will benefit nursing care, providing more effective assistance. One way to provide this care is by using health technologies as a strategy to help promote the health of this population.

In the area of anxiety, there are several scales that support the provision of assessment, treatment and patient care services, such as the State-Trait Anxiety Inventory (STAI)^(11)^, which was selected for use in the present study to measure the anxiety level of mothers of children confirmed to have COVID-19.

The STAI is an inventory designed to measure people’s anxiety levels, making it a relevant health technology for identifying health changes. It consists of two scales: STAI-State and STAI-Trait. The first assesses anxiety based on a momentary reaction, being associated with adverse circumstances such as the COVID-19 pandemic, while the second is related to a more permanent aspect, being associated with how individuals cope with anxiety throughout their life^(12)^.

Investigating the anxiety levels of mothers of children with COVID-19 in the first year of life using the STAI may provide information to identify the anxiety levels experienced by mothers following their children’s COVID-19 diagnosis. Furthermore, it will contribute to the planning of early interventions and health promotion targeted at this population, thus favoring safe and humane nursing care and preventing harm to the mother-child dyad.

## OBJECTIVES

To assess the anxiety level of mothers of children who had COVID-19 in a city in northeastern Brazil using the STAI.

## METHODS

### Ethical aspects

The data of study participants, as well as their telephone contact information, contained in the e-SUS *Notifica* system, were authorized and made available by the Epidemiological Surveillance Unit of the municipality of Fortaleza after submission and approval of the study by the *Universidade Federal do Ceará* Research Ethics Committee, through the *Plataforma Brasil*. The study was approved under Opinion 5,777,560, based on Resolution 466/12 of the Brazilian Ministry of Health. The study followed the General Data Protection Law rules, respecting principles such as necessity, purpose, and transparency. Researchers guaranteed the anonymity and protection of the information provided, and participants obtained explicit consent during calls.

### Study design, period, and location

This is a descriptive, cross-sectional, quantitative study, guided by the Strengthening the Reporting of OBservational studies in Epidemiology checklist.

Data collection was carried out from May to July 2023 in the municipality of Fortaleza, Ceará, Brazil. Fortaleza has a total area of 314,930 km^2^ and a population of 2,452,185 inhabitants. It is administratively regionalized, with the aim of reducing disparities between regions, into 12 Regional Departments, encompassing six Regional Health Coordination Offices, numbered I to VI, plus the Central Regional Department, totaling 121 neighborhoods^(13)^.

### Population or sample; inclusion and exclusion criteria

The study population consisted of mothers of children with COVID-19 in the first year of life, reported in the e-SUS *Notifica* system, from March 2020 to January 2021, residing in Fortaleza, Ceará.

The e-SUS *Notifica* system is a tool for registering notifications of suspected and confirmed cases of the novel coronavirus (COVID-19), made available by the Unified Health System Informatics Department throughout the national territory, allowing the import and export of this data for tabulation and integration of this system with other health systems^(14)^.

In order to select the participants who comprised the research sample, mothers of children aged 0 to 1 year old diagnosed with COVID-19 between March 2020 and January 2021, provided they had a telephone contact number registered in the e-SUS database and resided in the municipality of Fortaleza, Ceará, were included. Mothers with duplicate records in the information system were excluded.

Moreover, 289 mothers of children were identified in the e-SUS *Notifica* system, and 288 mothers met the inclusion criteria. However, 14 were excluded from this number due to duplicate records. From a sample of 274 mothers, 50 refused to participate; 69 did not answer calls; 73 had inconsistent data (date of birth); and 48 had no contact phone number. A total of 34 mothers participated in the research.

### Study protocol

Independent variables were extracted from the e-SUS *Notifica* database and the questionnaire administered to mothers, including maternal sociodemographic variables (age, origin, marital status, education level, race, family income). Concerning dependent variables, the anxiety levels presented by mothers of children who had COVID-19 in the first year of life were extracted from STAI assessment.

Data collection was carried out by the principal investigator and two other members of the research group to which the study belongs, from May to July 2023, through telephone contact with the mothers included in the study. The calls were made using a smartphone and lasted approximately 20 minutes, using SIM cards acquired exclusively for the research. Calls were conducted in the morning, from 9:00 to 11:00, and in the afternoon, from 15:00 to 17:00, Monday to Friday. It is important to note that the authors who collected the data received a standard operating procedure containing all the instructions about the research and were trained on how to proceed during the calls, as well as in instrument application.

Data were collected through telephone contact with the mothers included in the study. The calls were recorded using the Automatic Call Recorder application to document the mother’s agreement to participate in the research, including the Informed Consent Form. After participants’ agreement through the Post-Informed Consent Form, a questionnaire containing socioeconomic, demographic, clinical, and epidemiological variables, and the STAI to measure mothers’ anxiety levels, were administered during the call. The instruments were completed with the responses provided by participants.

The STAI was created as a self-report instrument composed of two scales, each containing 20 items corresponding to trait anxiety and state anxiety, totaling 40 items. The state scale requires participants to describe how they feel at a given moment, and the trait scale asks participants to indicate how they generally feel. The STAI is a Likert-type scale containing 4 points, but with distinct responses for each scale. On the trait anxiety scale, the probabilities of responses are: almost never (1); sometimes (2); often (3); almost always (4). On the state anxiety scale, the response options are: not at all (1); a little bit (2); moderately so (3); very much so (4).

### Analysis of results and statistics

In order to interpret the results and calculate the total STAI scores, a score is assigned to each answer given to each question in the instrument. Scores for positive questions are reversed so that if participants mark option 4, the value 1 will be assigned in the coding, if they answer 3, it will be 2, if they answer 2, it will be 3, and if they answer 4, it will be 1. Thus, the author describes positive (1, 6, 7, 10, 13, 16, 19) and negative (2, 3, 4, 5, 8, 9, 11, 12, 14, 15, 17, 18, 20) responses in the STAI-Trait. In the STAI-State, positive responses are 1, 2, 5, 8, 10, 11, 15, 16, 19, 20, and negative responses are 3, 4, 6, 7, 9, 12, 13, 14, 17, 18^(15)^.

In order to classify anxiety levels, STAI scores were subdivided into three percentiles, and the range of scores that comprised each percentile was decided after normalization analysis of collected data: percentile < 25 (low anxiety): STAI-Trait (scores 23 to 35) and STAI-State (scores 22 to 35); percentile 25 to 75 (moderate anxiety): STAI-Trait (scores 36 to 62) and STAI-State (scores 36 to 53); and percentile > 75 (high anxiety): STAI-Trait (scores 63 to 69) and STAI-State (scores 54 to 63)^(16,17)^.

Study variables were first extracted from the e-SUS *Notifica* database, provided by the Epidemiological Surveillance Unit of the municipality of Fortaleza, and tabulated in Microsoft Office Excel version 2016. This included information on the child’s and mother’s names, the child’s date of birth, the year of notification and symptom onset, address with health regions, contact phone number, and clinical and epidemiological variables of the disease. Subsequently, information on maternal variables from the instrument applied to the mothers during telephone calls was added to the same spreadsheet.

Statistical analyses were performed using IBM^®^ SPSS^®^ Statistics 29, in which descriptive, analytical, inferential, and association tests were conducted. Descriptive statistics were used to analyze dependent and independent variables. For continuous variables, means, medians, and standard deviations were calculated. For categorical variables, frequencies were estimated. To verify the association among variables, the Fisher-Freeman-Halton exact test was used, which is suitable for comparing categorical variables with a category frequency < 5. For this purpose, continuous independent variables were categorized according to what is described in the literature.

Furthermore, the dependent variables (outcomes) were also categorized according to the level of anxiety (percentiles), with the 25^th^ percentile representing low anxiety, the 25^th^ to 75^th^ percentiles representing moderate anxiety, and the 100^th^ percentile representing high anxiety. A 95% Confidence Interval (95%CI) was provided, where relationships with a significance level of 5% (p < 0.05) were considered statistically significant. Some categorical variables, both dependent and independent, were also categorized using binary (dichotomous) values in order to assess the statistical significance of this association.

## RESULTS

In relation to the maternal age of the 34 mothers who comprised the sample, 52.94% (18) were in the age range of 30 years or older; 73.53% (25) considered themselves brown; 47.06% (16) were married; and 41.18% were single (14). Concerning education, 41.18% (14) of mothers stated that they had completed high school; 38.24% (13) had completed higher education; and 20.59% (7) had completed elementary school. Regarding income, 20.59% (7) reported that the family earned less than one minimum wage, while 41.18% (14) of mothers stated that they had an income between one and two minimum wages. Concerning origin, all mothers resided in the municipality of Fortaleza, but Regional VI concentrated 47.06% (16) of participants, contrasting with Regional I, with only 2.94%.

Concerning the epidemiological variables of mothers of children with COVID-19, 67.65% (23) were infected by the virus, with 46.43% (13) diagnosed by rapid test, 35.71% (10) by Reverse Transcriptase Polymerase Chain Reaction, and 17.86% (5) by serology. As for the presence of the disease at the same time as their child, 68% (17) of mothers gave an affirmative answer.

When assessing the anxiety levels of mothers of children with COVID-19 using the STAI-Trait and STAI-State scales, where the state scale describes how mothers felt when their children were confirmed with COVID-19 in 2020 or 2021 and the trait scale indicates how mothers generally feel, it was identified that most mothers presented total scores in the percentile range between 25 and 75, with 50% (17) for the STAI-Trait and equally 50% (17) for the STAI-State, characterizing moderate anxiety levels, according to Table 1.

**Table 1:** Anxiety levels of mothers of children with COVID-19 (N = 34) according to the State-Trait Anxiety Inventory, Fortaleza, Ceará, Brazil, 2023

Percentile interval (n= 34)	n(%)	Mean±SD
**STAI-Trait**		48.41±13.72
Percentile < 25 (low anxiety)	7(20.59)	
Percentile 25 - 75 (moderate anxiety)	17(50.00)	
Percentile > 75 (high anxiety)	10(29.41)	
**STAI-State**		44.94±11.37
Percentile < 25 (low anxiety)	8(23.53)	
Percentile 25 - 75 (moderate anxiety)	17(50.00)	
Percentile > 75 (high anxiety)	9(26.47)	

*SD - standard deviation; STAI - State-Trait Anxiety Inventory.*

The association between maternal characteristics and anxiety outcomes was established (Table 2). According to the association, no statistical significance was observed in relation to the STAI-Trait percentiles. Thus, age, race, marital status, education, income, and region of residence were not associated with increased anxiety levels in the study participants. However, regarding the association with the STAI-State percentiles, a relationship was found with education, highlighting that mothers with lower education levels presented higher anxiety levels.

**Table 2:** Distribution of the number of mothers (N = 34) by percentile intervals and p-values according to the association between the Trait-State-Trait Anxiety Inventory and the Trait-State-State Anxiety Inventory with maternal sociodemographic variables, Fortaleza, Ceará, Brazil, 2023

	STAI-Trait				STAI-State			
**Maternal variables**	**< 25**	**25- 75**	**>75**		**< 25**	**25- 75**	**>75**	
	**n(%)**	**n(%)**	**n(%)**	***p*** ^†^	**n(%)**	**n(%)**	**n(%)**	***p*** ^†^
**Age (years)**				0.288^‡^				0.999^‡^
15 - 29	5 (31.3)	6 (37.4)	5 (31.3)		4 (25.0)	8 (50.0)	4 (25.0)	
> 30	2 (11.1)	11 (61.1)	5 (27.8)		4 (22.2)	9 (50.0)	5 (27.8)	
**Race**				0.647^‡^				0.841^‡^
White	1 (12.5)	3 (37.5)	4 (50.0)		1 (12.5)	4 (50.0)	3 (37.5)	
Brown	6 (24.0)	13 (52.0)	6 (24.0)		7 (28.0)	12 (48.0)	6 (24.0)	
Black	0 (0.0)	1 (100.0)	0 (0.0)		0 (0.0)	1 (100.0)	0 (0.0)	
**Marital status**				0.248^‡^				0.388^‡^
Single	1 (7.1)	7 (50.0)	6 (42.9)		2 (14.3)	9 (64.3)	3 (21.4)	
Married	5 (31.3)	8 (50.0)	3 (18.8)		6 (37.5)	6 (37.5)	4 (25.0)	
Common-law marriage	1 (33.3)	2 (66.7)	0 (0.0)		0 (0.0)	1 (33.3)	2 (66.7)	
Legally separated	0 (0.0)	0 (0.0)	1 (100.0)		0 (0.0)	1 (100.0)	0 (0.0)	
**Education**				0.476^‡^				**0.035** ^‡^
Elementary school	1 (14.3)	3 (42.9)	3 (42.9)		1 (14.3)	1 (14.3)	5 (71.4)	
High school	4 (28.6)	5 (35.7)	5 (35.7)		5 (35.7)	8 (57.1)	1 (7.1)	
Higher education	2 (15.4)	9 (69.2)	2 (15.4)		2 (15.4)	8 (61.5)	3 (23.1)	
**Income (minimum wage)^§^**				0.599^‡^				0.164^‡^
< 1	1 (14.3)	3 (42.9)	3 (42.9)		1 (14.3)	1 (14.3)	5 (71.4)	
1 - 2	4 (28.6)	5 (35.7)	5 (35.7)		4 (28.6)	8 (57.1)	2 (14.3)	
2 ≥ 3	1 (25.0)	2 (50.0)	1 (25.0)		1 (25.0)	3 (75.0)	0 (0.0)	
> 4	1 (11.1)	7 (77.8)	1 (11.1)		2 (22.2)	5 (55.6)	2 (22.2)	

STAI - State-Trait Anxiety Inventory;

† p - significance level;

‡ Fisher’s exact test;

§ Current minimum wage = R$1,320.00, Brazil, 2023.

The association between maternal epidemiological variables and percentile intervals according to the STAI-Trait and STAI-State scales did not show statistical significance (p > 0.05), according to the data described in Table 3. Thus, the presence of COVID-19, the type of test performed, and the onset of the disease simultaneously with that of children were not associated with increased maternal anxiety levels.

**Table 3:** Distribution of the number of mothers (N = 34) by percentile intervals and p-values according to the association between the Trait-State-Trait Anxiety Inventory and the Trait-State-State Anxiety Inventory with maternal epidemiological variables, Fortaleza, Ceará, Brazil, 2023

Maternal variables	STAI-Trait				STAI-State			
	< 25	25 - 75	> 75		< 25	25 - 75	> 75	
	n(%)	n(%)	n(%)	*p* ^†^	n(%)	n(%)	n(%)	*p* ^†^
**Mother had COVID-19**				0.705^‡^				0.797^‡^
Yes	4 (17.4)	11 (47.8)	8 (34.8)		5 (21.7)	11 (47.8)	7 (30.4)	
No	3 (27.3)	6 (54.5)	2 (18.2)		3 (27.3)	6 (54.5)	2 (18.2)	
**Type of test run**				0.428^‡^				0.551^‡^
RT-PCR	1 (10.0)	7 (70.0)	2 (20.0)		2 (20.0)	4 (40.0)	4 (40.0)	
Serology	2 (40.0)	3 (60.0)	0 (0.0)		1 (20.0)	3 (60.0)	1 (20.0)	
Rapid test	4 (30.8)	5 (38.5)	4 (30.8)		4 (30.8)	7 (53.8)	2 (15.4)	
**Had COVID-19 at the same time as the child**				1.000^‡^				0.096^‡^
Yes	3 (17.6)	9 (52.9)	5 (29.4)		4 (23.5)	10 (58.8)	3 (17.6)	
No	1 (12.5)	4 (50.0)	3 (37.5)		1 (12.5)	2 (25.0)	5 (62.5)	

STAI - State-Trait Anxiety Inventory;

† p - significance level;

‡ Fisher’s exact test; RT-PCR - Reverse Transcriptase Polymerase Chain Reaction.

Considering the bivariate STAI-State outcome, represented by high anxiety (percentile >75) and having mild or moderate anxiety (percentile <75), it is demonstrated that the mother’s income, as well as her education level, shows a statistically significant association, according to Table 4.

**Table 4:** Bivariate outcome analysis of the State-Trait Anxiety Inventory, education level, mother’s income, and *p*-values, Fortaleza, Ceará, Brazil, 2023

Maternal variables	STAI-State		*p* ^†^
	Moderate or light anxiety (Percentile<75)	High anxiety (Percentile>75)	
	n(%)	n(%)	
**Education**			**0.010** ^‡^
Elementary school	2 (28.6)	5 (71.4)	
High school	13 (92.9)	1 (7.1)	
Higher education	10 (76.9)	3 (23.1)	
**Income (minimum wage)^§^**			**0.027** ^‡^
< 1	2 (28.6)	5 (71.4)	
1 - 2	12 (85.7)	2 (14.3)	
2 ≥ 3	4 (100.0)	0 (0.0	
> 4	7 (77.8)	2 (22.2)	

STAI - State-Trait Anxiety Inventory;

† p - significance level;

‡ Fisher’s exact test;

§ Current minimum wage - R$1,320.00, Brazil, 2023.

## DISCUSSION

This research focused on the anxiety of mothers of children with COVID-19 in the first year of life, specifically in 2020 and early 2021, in a capital city in northeastern Brazil, when the SARS-CoV-2 pandemic was at its most critical in terms of the number of cases and uncertainties in the field of science regarding therapies, interventions, and ways of caring.

The results showed a predominance of mothers of mixed race, married, and aged 30 or older. Participants’ mean age was similar to studies conducted in the United Kingdom^(18)^ and Canada^(19)^ during the pandemic. Furthermore, it was found that the mother’s age could be associated with fear related to childcare in the context of the pandemic, raising maternal anxiety levels^(18)^. Regarding maternal education, secondary education predominated, similar to research in Turkey with postpartum women, which obtained the same result^(20)^_._

Income was another relevant factor, with mothers earning an average of one to two minimum wages standing out. Similar results were reported in exploratory and observational research on the mental health of mothers of children with microcephaly during the pandemic in the city of Campina Grande, Paraíba, revealing that 56.9% of these women had a family income greater than one and less than three minimum wages^(21)^. In a longitudinal observational study, a Canadian cohort observed an increase in maternal depression and anxiety during the COVID-19 pandemic, associated with income disruption, difficulty balancing homeschooling, and challenges with childcare^(22)^.

Most mothers reside in Regional VI, a territory that concentrates the largest area of the municipality of Fortaleza, with high population density and areas of social vulnerability, which makes care and prevention actions with the child more worrying, causing fear and anxiety on the part of caregivers, especially the mother. In another study involving children with COVID-19, this territory concentrated the highest number of infected children^(23)^, as well as other aggravations, such as the predominance of mothers of children with microcephaly due to Zika virus^(24)^, characterizing an area at risk of infection in this territory for COVID-19 and other diseases.

Regarding epidemiological variables, most mothers were infected with the virus at the same time as their child, similar to a study of 300 babies treated in hospitals at health centers in Poland, where 57% were diagnosed with COVID-19 at the same time as another family member in the household^(25)^. This fact was confirmed by a scoping review that describes that the disease in children and adolescents is most frequently acquired through transmission between family members^(26)^.

Moderate anxiety was identified in mothers whose children were confirmed with COVID-19, both during the infection period and at the time of the interview, characterized by the STAI-State and STAI-Trait.

Therefore, a meta-analysis conducted to identify the prevalence of depression and anxiety symptoms in parents of children under 5 years of age during the COVID-19 pandemic showed an estimated 26.9% (95%CI: 21.3-33.4) for depression and 41.9% (95%CI: 26.7-58.8) for anxiety in mothers, with elevated symptoms in Europe and North America and among older mothers^(27)^. In research conducted in Turkey, 21.2% of mothers of children aged 3 to 6 years presented moderate to severe anxiety, but 0.3% justified the anxiety as being caused by COVID-19^(28)^.

The pandemic caused disturbances in maternal mental health, which can be intensified when the child is infected by the virus, generating even more anxiety and concern about the risks of the disease. Furthermore, the pandemic imposed negative repercussions on families, with sadness being one of the identified feelings. Concern for the health of family members and the fear of contracting or transmitting the virus triggered new habits and behaviors, becoming more intense in families where people with COVID-19 required longer hospital stays and intensive care^(29)^.

In Chile, the health crisis caused by COVID-19 had a negative impact on women’s mental health during their transition to motherhood, with more intense symptoms of depression and anxiety observed in the first postpartum months^(30)^. A study with 3,939 postpartum women from Brazil, Chile, Cyprus, Greece, Israel, Portugal, Spain, Turkey, and the United Kingdom found a prevalence of 26.6% of women who presented symptoms of anxiety during the pandemic, with those residing in Brazil being the most likely to develop depression and anxiety^(31)^.

When correlating maternal sociodemographic characteristics with the STAI-Trait, no statistical association was found; an association was only found with the STAI-State. Thus, mothers with low education and lower income presented elevated anxiety levels during the period when their children tested positive for COVID-19.

An Italian study that used the STAI to identify the impact of COVID-19 on 178 pregnant women showed that there was a relationship between the anxiety of pregnant women and the pandemic, and that the higher education level of these women was correlated with the STAI-State values^(32)^. Furthermore, in a study involving the Beck Anxiety Inventory application in postpartum women, 763 women with completed secondary education presented moderate to severe anxiety^(33)^. Another study conducted in Turkey, which also used the inventory to identify anxiety levels in parents of children suspected of having COVID-19, showed that, in addition to moderate anxiety and fear of the disease, increased educational attainment was associated with parents’ positive attitudes towards protective measures and knowledge about the disease^(8)^.

Given the complexity of maternal health for mothers and their children, factors impacting various dimensions of mental health, such as existing social and economic inequalities, can be intensified^(34)^. In a harmonized sample of low-income mothers in three cities in the United States, a strong association was identified between loss of income, jobs, and health insurance since the onset of COVID-19 cases with symptoms of depression and anxiety^(35)^.

A systematic review demonstrated that there is an association between high anxiety and income in pregnant and postpartum women^(36)^. A study with mothers of children with congenital Zika virus syndrome expressed anxiety due to concerns about income in the face of the pandemic scenario, which negatively impacts the routine of care and stimulation required for children^(37)^. Moreover, another study reaffirms the increase in anxiety related to low income in mothers of newborns hospitalized in intensive care^(38)^.

Thus, COVID-19 generated significant emotional fragility in maternal health, consequently affecting maternal actions aimed at childcare. Emphasizing the burden and impact on maternal care during the pandemic, data indicate increased anxiety and fear of the future^(37)^. The pandemic and its social effects exacerbated maternal suffering and anxiety, especially in cases where the child presented risk factors such as prematurity. The limitation of maternal presence in the hospital environment, coupled with the reduction of the support network, financial difficulties, and increased emotional burden, contributed to intensifying the impact on mothers^(39)^. Anxiety is a relevant research topic in the mother-baby context, as this feeling can generate negative repercussions on maternal and child health^(7)^.

Given this, the need for epidemiological studies capable of shedding light on the impacts of infection by the new coronavirus is understood, requiring interventions in maternal anxiety management, as well as educational activities and public policies directed at this population.

### Study limitations

As for study limitations, there was difficulty in collecting information due to difficult reading and a lack of data in the information system database, mainly because COVID-19, at the time of the study, was considered a new threat to human health. Another limitation concerns the acquisition of data by telephone contact, since, because the data collection was based on contacts registered in 2020, there was a large number of invalid and incorrect telephone numbers, and mothers who refused to participate in the research, justifying the sample size of interviewees, which consisted only of mothers from one Brazilian capital city.

### Contributions to nursing, health, or public policy

The results achieved in this study may contribute to the scientific framework, as well as motivate further research including the current post-pandemic reality, bringing benefits to maternal and child health and guiding strategies for health professionals, especially regarding nursing care and care management in the face of these mothers’ mental health situations, including maternal anxiety.

## CONCLUSIONS

From this study, it can be concluded that the majority of the mothers studied were 30 years of age or older, brown, married, had completed high school, and had an income between one and two minimum wages. Furthermore, most mothers were infected with COVID-19 during the same period as their children.

Regarding anxiety, mothers presented scores between the 25^th^ and 75^th^ percentiles on both the STAI-Trait and STAI-State scales, representing moderate anxiety levels in the context of children confirmed with COVID-19 in their first year of life. When analyzing possible associations between total STAI-State scores and the mother’s sociodemographic variables, factors such as education and income were described as having a direct relationship with maternal anxiety levels. In this sense, it was concluded that mothers with low education and lower income presented elevated anxiety levels during the period of their children’s COVID-19 confirmation.

Stressful events, such as COVID-19, have contributed to the triggering of fluctuating anxiety levels, impacting maternal mental health. Therefore, health promotion and prevention strategies should be offered, enabling the implementation of multidisciplinary care, especially nursing care, focused on the complaints and other particularities of this population, aiming to prevent or mitigate mental disorders.

## Data Availability

The research data are not available.
