# Supplementary material for: Anxiety in mothers of children with COVID-19 based on the State-Trait Anxiety Inventory
Source: Rev Bras Enferm. 2026 Jul 31;79:e20240643. doi: 10.1590/0034-7167-2024-0643 (PMC13427022; doi:10.1590/0034-7167-2024-0643)
Supplement: Translation

# Translation: Ansiedade de mães de crianças com COVID-19 a partir do Inventário de Ansiedade Traço-Estado

**DOI:** 10.1590/0034-7167-2024-0643pt

**Authors:** Nagila Lima Fontenele, Daisyanne Augusto de Sales Santos, Isabela Araújo Linhares Castro, Felipe Moreira de Paiva, Fabíola Chaves Fontoura, Natalia Rodrigues Oliveira, Rhaiany Kelly Lopes de Oliveira, Maria Vera Lúcia Moreira Leitão Cardoso

**Affiliations:** I Centro Universitário Christus, Unichristus. Fortaleza, Ceará, Brasil; II Universidade Federal do Ceará. Fortaleza, Ceará, Brasil; III Hospital Regional Tarcísio de Vasconcelos Maia. Mossoró, Rio Grande do Norte, Brasil; IV Universidade Federal do Rio Grande, Hospital Universitário, Empresa Brazileira de Serviços Hospitalares. Rio Grande, Rio Grande do Sul, Brasil

**Keywords:** Ansiedade, Mães, COVID-19, Criança, Enfermagem.

## Abstract

**Objetivos:**

avaliar o nível de ansiedade de mães de crianças que tiveram COVID-19 em uma cidade do nordeste do Brasil usando o Inventário de Ansiedade Traço-Estado.

**Métodos:**

transversal, com mães de crianças com COVID-19 no primeiro ano de vida, notificadas no sistema e-SUS Notifica de fevereiro de 2020 a janeiro de 2021. Coleta ocorreu via contato telefônico, com aplicação de formulário sobre variáveis sociodemográficas e epidemiológicas maternas, além do Inventário de Ansiedade Traço- Estado. Para associação entre variáveis, utilizou-se teste de exato de Fisher-Freeman-Halton.

**Resultados:**

ansiedade materna foi moderada (percentil entre 25 e 75) tanto para o Inventário de Ansiedade - Traço (50%) quanto para o Inventário de Ansiedade-Estado (50%). Houve significância estatística para ansiedade elevada, entre escolaridade (p=0,010), renda (p=0,027) e o Inventário de Ansiedade-Estado.

**Conclusões:**

mães com filhos confirmados para COVID-19 apresentaram níveis moderados de ansiedade, com elevação naquelas com baixa escolaridade e baixa renda.

## INTRODUÇÃO

A COVID-19, infecção causada pelo coronavírus SARS-CoV-2, responsável pela Síndrome Respiratória Aguda Grave^(1)^, disseminou-se rapidamente pelo mundo. Além dos efeitos imediatos relacionados aos sintomas clínicos e ao risco de morte, a pandemia provocou consequências persistentes, intensificadas pelos longos períodos de isolamento social. Esses fatores desencadearam diversos transtornos na população, com repercussões econômicas, sociais^(2)^ e, especialmente, na saúde mental^(3)^. Segundo dados da Organização Mundial da Saúde^(4)^, houve um aumento de 25% nos casos de sintomas de ansiedade em diversos países, afetando, de maneira mais expressiva, as mulheres, que se mostraram particularmente suscetíveis ao desenvolvimento de transtornos mentais durante esse período.

Os questionamentos sobre os impactos da COVID-19 na saúde materna e neonatal aumentaram ainda mais a ansiedade no período gestacional. Na Coréia do Sul, não somente a ansiedade, mas também sintomas de depressão, transtorno de estresse pós-traumático e ideação suicida foram significativamente maiores em gestantes infectadas^(5)^.

Ao se tornar mãe, o gerenciamento da saúde física e mental se torna cada vez mais desafiador e estressante, contribuindo para agravamento dos sintomas de ansiedade, o que foi evidenciado em estudo realizado em Israel, levando cerca de 80% das mães investigadas a relatar níveis leves a altos de ansiedade em decorrência da COVID-19^(6)^. Além disso, a pandemia favoreceu ocasionar implicações no vínculo entre o binômio mãe e filho no que se refere aos cuidados com o bebê e ao apego afetivo^(7)^.

Os níveis de ansiedade vivenciados pela mãe podem ser ainda maiores ao ser confirmado o diagnóstico de COVID-19 no filho. Estudo conduzido com pais de crianças suspeitas para COVID-19 em ambulatório de um hospital terciário em Ancara, Turquia, evidenciou nível de ansiedade moderado dos pais e alto nível de medo na transmissão do vírus^(8)^. Outro estudo, no Centro Médico da Universidade de Nebraska, realizou uma coorte observacional com 703 mães e revelou que as taxas de ansiedade materna, depressão, uso de medicamentos para condições psiquiátricas e histórico de depressão pós-parto aumentaram significativamente durante a COVID-19, considerando os partos entre março de 2020 e dezembro de 2021. Permaneceram em níveis elevados após esse período em comparação com os níveis antes da pandemia^(9)^.

A saúde mental comprometida pode ter consequências imediatas e de longo prazo para as mães e seus bebês, ressaltando a necessidade urgente de cuidados e intervenções direcionados^(10)^. Assim, tendo em vista a importância do papel materno no bem-estar da criança, a avaliação da ansiedade é uma medida preventiva e protetiva para a mulher que irá beneficiar o cuidado de enfermagem, proporcionando uma assistência mais efetiva. Uma das formas de se exercer esse cuidado é fazendo uso de tecnologias em saúde como estratégia para auxiliar na promoção da saúde dessa população.

Na temática da ansiedade, existem várias escalas que dão suporte à prestação dos serviços de avaliação, tratamento e assistência ao paciente, como o Inventário de Ansiedade Traço-Estado (IDATE)^(11)^, o qual foi selecionado para ser utilizado no presente estudo para mensurar o nível de ansiedade de mães de crianças confirmadas para COVID-19.

O IDATE é um inventário que objetiva medir os níveis de ansiedade das pessoas, sendo, pois, uma tecnologia em saúde relevante na identificação de alterações na saúde, e é composto por duas escalas: IDATE-Estado e IDATE-Traço. A primeira avalia a ansiedade a partir de uma reação momentânea, estando associada a circunstâncias adversas, como a pandemia de COVID-19, ao passo que a segunda está relacionada a um aspecto mais permanente, estando associado à forma do indivíduo de lidar com a ansiedade no decorrer da sua vida^(12)^.

Investigar o nível de ansiedade de mães de crianças com COVID-19 no primeiro ano de vida por meio do IDATE poderá fornecer informações para identificar os níveis de ansiedade vivenciados pela mãe a partir do diagnóstico de COVID-19 em seus filhos. Além disso, contribuirá no planejamento de intervenções precoces e de promoção da saúde direcionadas a essa população, o que favorece um cuidado de enfermagem seguro e humanizado, evitando prejuízos à díade mãe-filho.

## OBJETIVOS

Avaliar o nível de ansiedade de mães de crianças que tiveram COVID-19 em uma cidade do nordeste do Brasil usando o IDATE.

## MÉTODOS

### Aspectos éticos

Os dados dos participantes do estudo, como também o contato telefônico, contidos no sistema e-SUS Notifica foram autorizados e disponibilizados pela Célula de Vigilância Epidemiológica do município de Fortaleza após submissão e aprovação do estudo pelo Comitê de Ética em Pesquisa com Seres Humanos da Universidade Federal do Ceará, por meio da Plataforma Brasil. O estudo foi aprovado sob Parecer n^o^ 5.777.560, pautado na Resolução n^o^ 466/12 do Ministério da Saúde do Brasil. Seguiu as regras da Lei Geral de Proteção de Dados, respeitando princípios como necessidade, finalidade e transparência, sendo garantidos pelos pesquisadores o anonimato e proteção das informações disponibilizadas, além de consentimento explícito dos participantes durante as ligações.

### Desenho, período e local do estudo

Trata-se de estudo descritivo, transversal, quantitativo, norteado pela lista de verificação do *Strengthening the Reporting of Observational Studies in Epidemiology*.

A coleta de dados foi realizada nos meses de maio a julho de 2023 no município de Fortaleza, Ceará, Brasil. Fortaleza tem uma área total de 314.930 km^2^ e uma população de 2.452.185 de habitantes, estando regionalizada administrativamente, com o intuito de diminuir as disparidades entre as regiões, em 12 Secretarias Regionais, contemplando seis Coordenadorias Regionais de Saúde, para organização dos serviços de saúde, indo de I a VI, mais a Secretaria Regional Centro, contabilizando 121 bairros^(13)^.

### População ou amostra; critérios de inclusão e exclusão

A população do estudo consistiu em mães de crianças com COVID-19 no primeiro ano de vida, notificadas no e-SUS Notifica, no período de março de 2020 a janeiro de 2021, residentes em Fortaleza, Ceará.

O e-SUS Notifica é uma ferramenta para registro de notificação de casos suspeitos e confirmados do novo coronavírus (COVID-19), disponibilizada pelo Departamento de Informática do Sistema Único de Saúde em todo o território nacional, sendo possível a importação e exportação desses dados para tabulação e integração desse sistema com outros sistemas de saúde^(14)^.

A fim de selecionar as participantes que compuseram a amostra da pesquisa, incluíram-se mães de crianças na faixa etária de 0 a 1 ano de idade diagnosticada com COVID-19 no período de março de 2020 a janeiro de 2021, com contato telefônico registrado no banco de dados do e-SUS e residentes do município de Fortaleza, Ceará. Excluíram-se mães com registros duplicados no sistema de informação.

Foram identificadas 289 mães de crianças no e-SUS Notifica, e 288 mães contemplaram os critérios de inclusão. No entanto, desse quantitativo, 14 foram excluídas por estarem com registros duplicados. De uma amostra de 274 mães, 50 recusaram participação; 69 não atenderam as ligações; 73 possuíam dados inconsistentes (data de nascimento); e 48 possuíam telefone de contato inexistente. Um total de 34 mães participaram na pesquisa.

### Protocolo do estudo

As variáveis independentes foram extraídas a partir do banco de dados do e-SUS Notifica e do formulário aplicado com as mães, sendo contempladas as variáveis sociodemográficas maternas (idade, procedência, estado civil, nível de escolaridade, raça, renda familiar). Em relação às variáveis dependentes, os níveis de ansiedade apresentados pelas mães de crianças que tiveram COVID-19 no primeiro ano de vida foram extraídos a partir da avaliação utilizando o IDATE.

A coleta de dados foi realizada pela pesquisadora principal e por dois outros membros do grupo de pesquisa a que o estudo pertence, de maio a julho de 2023, através de contato telefônico com as mães incluídas no estudo. As ligações foram realizadas com a utilização de aparelho do tipo *smartphone*, e duraram, aproximadamente, 20 minutos, a partir de *chips* adquiridos exclusivamente para a pesquisa, sendo realizadas no período da manhã, das 09:00 às 11:00, e no período da tarde, das 15:00 às 17:00, de segunda a sexta-feira. Salienta-se que os autores que coletaram os dados receberam procedimento operacional padrão contendo todas as instruções sobre a pesquisa, e foram treinados sobre como proceder durante as ligações, bem como na aplicação dos instrumentos.

Os dados coletados por meio de contato telefônico com as mães incluídas no estudo. Foram gravados pelo aplicativo *Automatic Call Recorder* como forma de registrar a anuência da mãe em fazer parte da pesquisa, sendo gravado o Termo de Consentimento Livre e Esclarecido. Após a concordância da participante através do Termo de Consentimento Pós-Informado, foram aplicados, durante a ligação, o formulário, com variáveis socioeconômicas, demográficas, clínicas e epidemiológicas, e o IDATE, para mensurar os níveis de ansiedade presentes nas mães. Os instrumentos foram preenchidos com as respostas fornecidas pelas participantes.

O IDATE foi criado como um instrumento de autorrelato composto por duas escalas, cada uma contendo 20 itens correspondentes à ansiedade-traço e à ansiedade-estado, totalizando 40 itens. A escala estado requer que a participante descreva como se sente no dado momento, e a escala traço solicita às participantes que indiquem como geralmente se sentem. O IDATE é do tipo Likert, contendo 4 pontos, porém com respostas distintas para cada uma das escalas. Na escala de ansiedade-traço, as probabilidades de respostas são: quase nunca (1); às vezes (2); frequentemente (3); quase sempre (4). Já na escala de ansiedade-estado, as opções de respostas são: não absolutamente (1); um pouco (2); bastante (3); muitíssimo (4).

### Análise dos resultados e estatística

Com a finalidade de interpretar os resultados e calcular os escores totais do IDATE, é determinada a pontuação à cada resposta dada para cada pergunta presente no instrumento. Escores para perguntas de caráter positivo são invertidos, de tal maneira que se o participante marcar a opção 4, será atribuído o valor 1 na codificação, se responder 3, será 2, se responder 2, será 3, e se responder 4, será 1. Com isso, o autor descreve no IDATE-Traço as respostas positivas (1, 6, 7, 10, 13, 16, 19) e negativas (2, 3, 4, 5, 8, 9, 11, 12, 14, 15, 17, 18, 20). No IDATE-Estado, as respostas positivas são 1, 2, 5, 8, 10, 11, 15, 16, 19, 20, e as negativas são 3, 4, 6, 7, 9, 12, 13, 14, 17, 18^(15)^.

A fim de classificar os níveis de ansiedade, os escores do IDATE foram subdivididos em três percentis, e o intervalo desses escores que compuseram cada percentil foi decidido após análise da normalização dos dados coletados: percentil < 25 (ansiedade baixa): IDATE-Traço (escores de 23 a 35) e IDATE-Estado (escores de 22 a 35); percentil 25 a 75 (ansiedade moderada): IDATE-Traço (escores de 36 a 62) e IDATE-Estado (escores de 36 a 53); e percentil > 75 (ansiedade elevada): IDATE-Traço (escores de 63 a 69) e IDATE-Estado (escores de 54 a 63)^(16,17)^.

As variáveis do estudo, primeiramente, foram extraídas do banco de dados e-SUS Notifica e disponibilizados pela Célula de Vigilância Epidemiológica do município de Fortaleza, tabulados no programa *Microsoft Office Excel,* versão 2016, com informações do nome da criança e da mãe, data de nascimento da criança, ano de notificação e de início de sintomas, endereço com regiões de saúde, telefone de contato, e variáveis clínicas e epidemiológicas da doença. Em seguida, nessa mesma planilha, foi adicionada informação das variáveis maternas do instrumento aplicado com as mães durante ligações telefônicas.

As análises estatísticas foram realizadas utilizando o programa *IBM^®^ SPSS^®^ Statistics* 29, no qual foram realizados testes de estatística descritiva, analítica, inferencial e de associação. Utilizou-se estatística descritiva para análise das variáveis dependentes e independentes. Para variáveis contínuas, utilizou-se o cálculo de médias, medianas e desvio padrão. Para as variáveis categóricas, estimaram-se as frequências. Para verificar a relação de associação entre as variáveis, foi utilizado o teste exato de Fisher-Freeman-Halton, adequado para comparar variáveis categóricas e que apresentam uma frequência de categorias < 5. Para tanto, as variáveis independentes contínuas foram categorizadas de acordo com o descrito na literatura.

Além disso, as variáveis dependentes (desfechos) também foram categorizadas de acordo com o nível de ansiedade (percentis), representando o percentil 25 como ansiedade baixa, os percentis 25 a 75, como ansiedade moderada, e o percentil 100, como ansiedade elevada. Representou-se um Intervalo de Confiança de 95% (IC95%), em que foram consideradas como estatisticamente significantes as relações com nível de significância de 5% (p< 0,05). Algumas variáveis categóricas, dependentes e independentes, foram categorizadas também em valores binários (dicotômicos), a fim de avaliar a significância estatística dessa associação.

## RESULTADOS

Em relação à idade materna das 34 mães que compuseram a amostra, 52,94% (18) encontravam-se na faixa etária igual ou maior que 30 anos; 73,53% (25) se consideraram parda; 47,06% (16) possuíam o estado civil casada; e 41,18% possuíam o estado civil solteira (14). No que concerne à escolaridade, 41,18% (14) das mães afirmaram ter concluído o ensino médio; 38,24% (13) concluíram o ensino superior; e 20,59% (7) concluíram o ensino fundamental. Em relação à renda, 20,59% (7) relataram que a família ganhava menos de um salário mínimo, enquanto 41,18% (14) das mães afirmaram possuir renda entre um e dois salários mínimos. Quanto à procedência, todas as mães residiam no município de Fortaleza, contudo a Regional VI concentrou 47,06% (16) das participantes, contrapondo-se à Regional I, com apenas 2,94%.

Em relação às variáveis epidemiológicas de mães de crianças com COVID-19, 67,65% (23) foram infectadas pelo vírus, sendo 46,43% (13) diagnosticadas com teste rápido, 35,71% (10), com Reação em Cadeia da Polimerase via Transcriptase Reversa, e 17,86% (5), com sorologia. Quanto à presença da doença no mesmo período do filho, 68% (17) das mães deram resposta afirmativa.

Ao avaliar os níveis de ansiedade das mães das crianças com COVID-19 a partir do IDATE-Traço e IDATE-Estado, em que a escala estado descreve como as mães se sentiram quando seus filhos foram confirmados com COVID-19 em 2020 ou 2021 e a escala traço indica como as mães geralmente se sentem, identifica-se que a maioria das mães apresentou escores totais no intervalo de percentil entre 25 e 75, sendo 50% (17) para o IDATE-Traço e igualmente 50% (17) para o IDATE-Estado, caracterizando níveis moderados de ansiedade, de acordo com a Tabela 1.

**Tabela 1:** Níveis de ansiedade das mães de crianças com COVID-19 (N = 34) segundo o Inventário de Ansiedade Traço-Estado, Fortaleza, Ceará, Brasil, 2023

Intervalos de percentis (n= 34)	n(%)	Média±DP
**IDATE-Traço**		48,41±13,72
Percentil < 25 (ansiedade baixa)	7(20,59)	
Percentil 25 - 75 (ansiedade moderada)	17(50,00)	
Percentil > 75 (ansiedade elevada)	10(29,41)	
**IDATE-Estado**		44,94±11,37
Percentil < 25 (ansiedade baixa)	8(23,53)	
Percentil 25 - 75 (ansiedade moderada)	17(50,00)	
Percentil > 75 (ansiedade elevada)	9(26,47)	

*DP - desvio padrão; IDATE - Inventário de Ansiedade Traço-Estado.*

Estabeleceu-se a associação entre as características da mãe e os desfechos de ansiedade (Tabela 2). De acordo com a associação, observou-se que não houve significância estatística em relação aos percentis do IDATE-Traço. Assim, a idade, raça, estado civil, escolaridade, renda e região de residência não estiveram associadas ao aumento dos níveis de ansiedade nas participantes do estudo. Entretanto, no que se refere à associação com os percentis do IDATE-Estado, encontrou-se relação com escolaridade, destacando que mães com menor nível de escolaridade apresentaram maior nível de ansiedade.

**Tabela 2:** Distribuição do número de mães (N = 34) por intervalos de percentis e valores de *p* segundo a associação entre o Inventário de Ansiedade Traço-Estado-Traço e o Inventário de Ansiedade Traço-Estado-Estado com variáveis sociodemográficas maternas, Fortaleza, Ceará, Brasil, 2023

	IDATE-Traço				IDATE-Estado			
**Variáveis maternas**	**< 25**	**25- 75**	**>75**		**< 25**	**25- 75**	**>75**	
	**n(%)**	**n(%)**	**n(%)**	***p*** ^†^	**n(%)**	**n(%)**	**n(%)**	***p*** ^†^
**Idade (anos)**				0,288^‡^				0,999^‡^
15 - 29	5 (31,3)	6 (37,4)	5 (31,3)		4 (25,0)	8 (50,0)	4 (25,0)	
> 30	2 (11,1)	11 (61,1)	5 (27,8)		4 (22,2)	9 (50,0)	5 (27,8)	
**Raça**				0,647^‡^				0,841^‡^
Branca	1 (12,5)	3 (37,5)	4 (50,0)		1 (12,5)	4 (50,0)	3 (37,5)	
Parda	6 (24,0)	13 (52,0)	6 (24,0)		7 (28,0)	12 (48,0)	6 (24,0)	
Negra	0 (0,0)	1 (100,0)	0 (0,0)		0 (0,0)	1 (100,0)	0 (0,0)	
**Estado civil**				0,248^‡^				0,388^‡^
Solteira	1 (7,1)	7 (50,0)	6 (42,9)		2 (14,3)	9 (64,3)	3 (21,4)	
Casada	5 (31,3)	8 (50,0)	3 (18,8)		6 (37,5)	6 (37,5)	4 (25,0)	
União consensual	1 (33,3)	2 (66,7)	0 (0,0)		0 (0,0)	1 (33,3)	2 (66,7)	
Separada judicialmente	0 (0,0)	0 (0,0)	1 (100,0)		0 (0,0)	1 (100,0)	0 (0,0)	
**Escolaridade**				0,476^‡^				**0,035** ^‡^
Fundamental	1 (14,3)	3 (42,9)	3 (42,9)		1 (14,3)	1 (14,3)	5 (71,4)	
Médio	4 (28,6)	5 (35,7)	5 (35,7)		5 (35,7)	8 (57,1)	1 (7,1)	
Superior	2 (15,4)	9 (69,2)	2 (15,4)		2 (15,4)	8 (61,5)	3 (23,1)	
**Renda (salário mínimo)** ^§^				0,599^‡^				0,164^‡^
< 1	1 (14,3)	3 (42,9)	3 (42,9)		1 (14,3)	1 (14,3)	5 (71,4)	
1 - 2	4 (28,6)	5 (35,7)	5 (35,7)		4 (28,6)	8 (57,1)	2 (14,3)	
2 ≥ 3	1 (25,0)	2 (50,0)	1 (25,0)		1 (25,0)	3 (75,0)	0 (0,0)	
> 4	1 (11,1)	7 (77,8)	1 (11,1)		2 (22,2)	5 (55,6)	2 (22,2)	

IDATE - Inventário de Ansiedade Traço-Estado;

† p - nível de significância;

‡ Teste exato de Fisher;

§ Salário mínimo vigente = R$1.320,00, Brasil, 2023.

A associação entre as variáveis epidemiológicas maternas e os intervalos de percentis de acordo com o IDATE-Traço e IDATE-Estado não apresentou significância estatística (p> 0,05), de acordo com os dados descritos na Tabela 3. Assim, a presença de COVID-19, o tipo de teste realizado e o surgimento da doença simultaneamente à do filho não estiveram associados à elevação nos níveis de ansiedade da mãe.

**Tabela 3:** Distribuição do número de mães (N = 34) por intervalos de percentis e valores de *p* segundo a associação entre o Inventário de Ansiedade Traço-Estado-Traço e o Inventário de Ansiedade Traço-Estado-Estado com variáveis epidemiológicas maternas, Fortaleza, Ceará, Brasil, 2023

Variáveis Maternas	IDATE-Traço				IDATE-Estado			
	< 25	25 - 75	> 75		< 25	25 - 75	> 75	
	n(%)	n(%)	n(%)	*p* ^†^	n(%)	n(%)	n(%)	*p* ^†^
**Mãe teve COVID-19**				0,705^‡^				0,797^‡^
Sim	4 (17,4)	11 (47,8)	8 (34,8)		5 (21,7)	11 (47,8)	7 (30,4)	
Não	3 (27,3)	6 (54,5)	2 (18,2)		3 (27,3)	6 (54,5)	2 (18,2)	
**Tipo de teste realizado**				0,428^‡^				0,551^‡^
RT-PCR	1 (10,0)	7 (70,0)	2 (20,0)		2 (20,0)	4 (40,0)	4 (40,0)	
Sorologia	2 (40,0)	3 (60,0)	0 (0,0)		1 (20,0)	3 (60,0)	1 (20,0)	
Teste rápido	4 (30,8)	5 (38,5)	4 (30,8)		4 (30,8)	7 (53,8)	2 (15,4)	
**Teve COVID-19 na mesma época que o filho**				1,000^‡^				0,096^‡^
Sim	3 (17,6)	9 (52,9)	5 (29,4)		4 (23,5)	10 (58,8)	3 (17,6)	
Não	1 (12,5)	4 (50,0)	3 (37,5)		1 (12,5)	2 (25,0)	5 (62,5)	

*IDATE - Inventário de Ansiedade Traço-Estado; ^†^p - nível de significância; ^‡^Teste exato de Fisher; RT-PCR - Reação em Cadeia da Polimerase via Transcriptase Reversa.*

Considerando o desfecho IDATE-Estado bivariado, representado por ansiedade elevada (percentil>75) e ter ansiedade leve ou moderada (percentil<75), demonstra-se que a renda da mãe, bem como a sua escolaridade, apresenta associação estatisticamente significante, de acordo com a Tabela 4.

**Tabela 4:** Análise com desfecho bivariado do Inventário de Ansiedade Traço-Estado-Estado, escolaridade, renda da mãe e valores de *p,* Fortaleza, Ceará, Brasil, 2023

Variáveis maternas	IDATE-Estado		*p* ^†^
	Ansiedade leve ou moderada (Percentil<75)	Ansiedade elevada (Percentil>75)	
	n(%)	n(%)	
**Escolaridade**			**0,010** ^‡^
Fundamental	2 (28,6)	5 (71,4)	
Médio	13 (92,9)	1 (7,1)	
Superior	10 (76,9)	3 (23,1)	
**Renda (salário mínimo)** ^§^			**0,027** ^‡^
< 1	2 (28,6)	5 (71,4)	
1 - 2	12 (85,7)	2 (14,3)	
2 ≥ 3	4 (100,0)	0 (0,0	
> 4	7 (77,8)	2 (22,2)	

IDATE - Inventário de Ansiedade Traço-Estado; ^†^p - nível de significância;

‡ Teste exato de Fisher;

§ Salário mínimo vigente - R$1.320,00, Brasil, 2023.

## DISCUSSÃO

A presente pesquisa enfocou o tema ansiedade das mães de crianças com COVID-19 no primeiro ano de vida, especificamente no ano de 2020 e início de 2021, em uma capital do nordeste brasileiro, quando a pandemia de SARS-CoV-2 estava nos momentos mais críticos em número de casos, incertezas no campo da ciência quanto às terapêuticas, intervenções e modo de cuidar.

Os resultados mostraram predominância de mães da cor parda, casada e com idade maior ou igual a 30 anos. A média de idade das participantes foi semelhante a estudos realizados no Reino Unido^(18)^ e no Canadá^(19)^ durante a pandemia. Além disso, constatou-se que a idade da mãe poderia estar associada ao medo relacionado aos cuidados com os filhos diante do contexto da pandemia, elevando os níveis de ansiedade materna^(18)^. No que se refere à escolaridade materna, preponderou o ensino médio, similar a pesquisa da Turquia com mulheres no pós-parto, quando se obteve o mesmo resultado^(20)^_._

A renda foi outro fator relevante, destacando-se as mães com média de um a dois salários mínimos. Resultados semelhantes ao descrito foram relatados em pesquisa exploratória e observacional sobre saúde mental de mães de crianças com microcefalia durante a pandemia, na cidade de Campina Grande, Paraíba, revelando que 56,9% dessas mulheres possuíam renda familiar maior que um e menor que três salários mínimos^(21)^. Em estudo observacional longitudinal, uma coorte canadense observou aumento de depressão materna e ansiedade durante a pandemia de COVID-19, sendo associada à interrupção de renda, à dificuldade em equilibrar a educação em casa e às dificuldades com o cuidado com o filho^(22)^.

A maioria das mães são residentes da Regional VI, território que concentra maior área do município de Fortaleza, com grande densidade populacional e áreas de vulnerabilidade social, o que torna mais preocupante as ações de cuidado e prevenção com a criança, ocasionando medo e ansiedade por parte dos cuidadores, em especial da mãe. Em outro estudo envolvendo crianças com COVID-19, esse território concentrou o maior número de infectados^(23)^, como também outros agravos, como a predominância de mães de crianças com microcefalia por vírus Zika^(24)^, caracterizando uma área de risco de infecção nesse território para COVID-19 e outras doenças.

Em relação às variáveis epidemiológicas, a maioria das mães foi infectada pelo vírus no mesmo período que o filho, ocorrendo similaridade com estudo de 300 bebês atendidos em hospitais de centros de saúde da Polônia, onde 57% foram diagnosticados com COVID-19 no mesmo período com outro familiar da residência^(25)^. Tal fato foi confirmado por revisão de escopo que descreve que a doença em crianças e adolescentes é adquirida com maior frequência pela transmissão entre familiares^(26)^_._

Identificou-se ansiedade moderada nas mães cujos filhos foram confirmados com COVID-19, tanto durante o período da infecção quanto no momento da entrevista, caracterizada pelo IDATE-Estado e IDATE-Traço.

Logo, meta-análise realizada para identificar a prevalência de sintomas de depressão e ansiedade em pais de crianças menores de 5 anos durante a pandemia de COVID-19 evidenciou uma estimativa de 26,9% (IC95%: 21,3-33,4) para depressão e 41,9% (IC95%: 26,7-58,8) para ansiedade nas mães, com sintomas elevados na Europa e América do Norte e entre mães com maior idade^(27)^. Em pesquisa desenvolvida na Turquia, 21,2% das mães de crianças entre 3 e 6 anos apresentaram ansiedade moderada a grave, mas 0,3% justificaram a ansiedade motivada pela COVID-19^(28)^.

A pandemia ocasionou transtornos na saúde mental materna, podendo ser intensificados quando a criança é infectada pelo vírus, gerando ainda mais ansiedade e preocupação com os riscos da doença. Ademais, a pandemia impôs repercussões negativas às famílias, sendo a tristeza um dos sentimentos identificados. A preocupação com a saúde dos familiares e o medo de contrair o vírus ou transmiti-lo desencadearam novos hábitos e comportamentos, tornando-se mais intensa nas famílias em que a pessoa com COVID-19 necessitou de maior tempo de internação e cuidados intensivos^(29)^_._

No Chile, a crise sanitária ocasionada pela COVID-19 teve um impacto negativo na saúde mental das mulheres durante sua transição para maternidade, sendo observados sintomas mais intensos de depressão e ansiedade nos primeiros meses pós-parto^(30)^. Estudo com 3.939 puérperas do Brasil, Chile, Chipre, Grécia, Israel, Portugal, Espanha, Turquia e Reino Unido verificou prevalência de 26,6% de mulheres que apresentaram sintomas de ansiedade durante a pandemia, sendo as residentes no Brasil as mais propensas a desenvolver depressão e ansiedade^(31)^.

Ao correlacionar características sociodemográficas maternas com o IDATE-Traço, não houve associação estatística, ocorrendo associação apenas com o IDATE-Estado. Assim, as mães com baixa escolaridade e menor renda apresentaram níveis de ansiedade elevados durante o período de confirmação dos filhos com COVID-19.

Estudo italiano que usou o IDATE a fim de identificar o impacto da COVID-19 em 178 grávidas evidenciou que houve relação entre a ansiedade das gestantes e a pandemia, e que a maior escolaridade dessas mulheres teve correlação com os valores do IDATE-Estado^(32)^. Além disso, em estudo que envolveu a aplicação do Inventário de Ansiedade de Beck em puérperas, 763 mulheres com ensino médio completo apresentaram ansiedade de moderada a grave^(33)^. Outro estudo realizado na Turquia, que também utilizou o inventário para identificar os níveis de ansiedade de pais com filhos com suspeita de COVID-19, evidenciou que, além da ansiedade moderada e medo da doença, o aumento do nível de escolaridade esteve associado à atitude positiva dos pais em relação às medidas de proteção e ao conhecimento sobre a doença^(8)^.

Diante da complexidade da saúde materna para mães e seus filhos, os fatores que impactam as várias dimensões da saúde mental, como as desigualdades sociais e econômicas existentes, podem ser intensificados^(34)^. Em uma amostra harmonizada de mães com baixa renda em três cidades dos Estados Unidos, identificou-se grande associação entre a perda de renda, empregos e seguro de saúde desde o início dos casos de COVID-19 com os sintomas de depressão e ansiedade^(35)^.

Revisão sistemática demonstrou que existe associação entre ansiedade elevada e renda em mulheres grávidas e puérperas^(36)^. Estudo com mães de crianças com síndrome congênita pelo vírus Zika expressou ansiedade por preocupação com a renda diante do cenário de pandemia, o que impacta negativamente a rotina de cuidado e estimulação exigida para a criança^(37)^. Somado a isso, outra pesquisa reafirma o aumento da ansiedade relacionado à baixa renda em mães de recém-nascidos internados em terapia intensiva^(38)^.

Desse modo, a COVID-19 gerou uma grande fragilidade emocional na saúde materna, afetando, consequentemente, as ações maternas voltadas ao cuidado à criança. Ao enfatizar a sobrecarga e o impacto na prestação do cuidado materno durante a pandemia, dados indicam elevação da ansiedade e medo do futuro^(37)^. A pandemia e seus efeitos sociais agravaram o sofrimento e a ansiedade materna, especialmente em casos em que a criança apresentava fatores de risco como a prematuridade. A limitação da presença materna no ambiente hospitalar, somada à redução da rede de apoio, às dificuldades financeiras e ao aumento da carga emocional, contribuiu para intensificar o impacto sobre as mães^(39)^. A ansiedade é um tema relevante de pesquisa no âmbito mãe-bebê, pois esse sentimento pode gerar repercussões negativas na saúde materna-infantil^(7)^.

Diante disso, compreende-se a necessidade de estudos epidemiológicos capazes de lançar luz sobre os impactos da infecção pelo novo coronavírus, sendo necessárias intervenções no manejo da ansiedade materna, bem como atividades educativas e políticas públicas direcionadas a esse público.

### Limitações do estudo

Em relação às limitações do estudo, houve dificuldade na coleta de informações devido à leitura difícil e à carência de dados no banco do sistema de informação, principalmente pelo fato de a COVID-19, no momento da realização estudo, ser considerada um novo agravo à saúde humana. Outra limitação diz respeito à aquisição dos dados por contato telefônico, pois, em razão da coleta ter sido a partir de contatos registrados em 2020, ocorreu grande número de telefones inválidos, incorretos e de mães que se recusaram a participar da pesquisa, justificando o número amostral de entrevistadas, o qual foi composto apenas por mães de uma capital brasileira.

### Contribuições para a área da enfermagem, saúde ou políticas públicas

Os resultados alcançados neste estudo poderão contribuir para o arcabouço científico, além de motivar a realização de novas pesquisas incluindo a realidade atual de pós-pandemia, trazendo benefícios para a saúde materna-infantil e direcionamento de estratégias para profissionais de saúde, principalmente no que se refere aos cuidados de enfermagem e gerenciamento do cuidado diante das situações de saúde mental dessas mães, incluindo a ansiedade materna.

## CONCLUSÕES

A partir deste estudo, pôde-se concluir que a maioria das mães estudadas apresentou idade igual ou maior que 30 anos, cor parda, casada, ensino médio completo e renda entre um e dois salários mínimos. Além disso, a maioria das mães foi infectada pela COVID-19 no mesmo período dos filhos.

Em relação à ansiedade, apresentaram escores entre os intervalos de percentis 25 a 75, tanto no IDATE-Traço quanto no IDATE-Estado, representando níveis moderados de ansiedade diante de um contexto de filhos confirmados com COVID-19 no primeiro ano de vida. Ao se analisar possíveis associações entre os escores totais do IDATE-Estado e as variáveis sociodemográficas da mãe, fatores como escolaridade e renda foram descritos como tendo relação direta com os níveis de ansiedade materna. Nesse sentido, concluiu-se que mães com baixa escolaridade e menor renda apresentaram níveis de ansiedade elevados durante o período de confirmação dos filhos com COVID-19.

Eventos estressores, como a COVID-19, contribuíram para o desencadeamento de oscilação de níveis de ansiedade, impactando a saúde mental materna. Com isso, estratégias de promoção e de prevenção à saúde devem ser ofertadas, possibilitando a implementação de cuidados multidisciplinares, em especial de enfermagem, voltados às queixas e outras particularidades dessa população, objetivando prevenir ou mitigar transtornos mentais.

## Data Availability

Os dados de pesquisa não estão disponíveis.
